# Analysis of critical nutrients in ketogenic diets for adults based on optimized hypothetical meal plans

**DOI:** 10.3389/fnut.2026.1829975

**Published:** 2026-09-11

**Authors:** Marc Assmann, Isabel Albrecht, Tobias Fischer

**Affiliations:** Center for Nutrition & Therapy (NuT), University of Applied Sciences Muenster, Muenster, Germany

**Keywords:** adults, critical nutrients, ketogenic diet, ketogenic nutrition, micronutrient deficiencies, micronutrients

## Abstract

**Introduction:**

Ketogenic diets are used in various clinical and non-clinical contexts in adults. Due to the high degree of restriction in certain ketogenic diet variations, there is a potential risk of insufficient micronutrient intake and systematic analyses of nutrient supply in adults remain sparse.

**Methods:**

Optimized, hypothetical daily ketogenic meal plans were created for adults aged 19 to over 65 years, with ketogenic ratios of 3:1, 2:1, and 1:1. The analysis was carried out separately for four age groups. Nutrient calculations were based on the German Nutrient Database using the PRODI® nutrition software (Nutri-Science GmbH, Germany). The calculated micronutrient intakes were compared with age-related national reference values for each age group. Supply < 95% was considered as potentially critical.

**Results:**

Relevant deficiencies were observed in all age groups, particularly in fiber, vitamin D, several B vitamins, potassium, zinc and fluoride. As restrictiveness decreased, intake levels improved, with the 1:1 ratio showing the highest overall micronutrient density. Regardless of the ratio, vitamin D, fiber and fluoride remained potentially critical in all age groups.

**Conclusion:**

Strict ketogenic ratios carry a high risk of micronutrient deficiencies. Although liberal ratios improve supply, there are still structural nutrient restrictions. Therefore, careful food selection and nutritional therapy support remain essential.

## 1 Introduction

The use of ketogenic diets (KD) is increasing in both clinical and non-clinical contexts among adults. In addition to their role in pharmacoresistant epilepsy, they are also administered for metabolic diseases such as obesity and type 2 diabetes mellitus, as part of oncological therapy and for weight loss or optimized performance (1, 2). Furthermore, there has been an increasing amount of discussion in recent years about the use of ketogenic diets to treat psychiatric disorders (2, 3). Clinical consensus reports and observational studies show adults sometimes initiate and follow KDs over long periods of time, raising questions about their long-term nutritional adequacy and micronutrient supply (4, 5). The KD is a high-fat, very low-carbohydrate, moderate-protein diet that aims to put the metabolism into a ketotic state. Significantly reducing carbohydrate intake lowers glucose and insulin levels promotes the use of fat for energy supply through increased β-oxidation. In addition, the resulting overload of the citric acid cycle leads to increased formation of the ketone bodies acetoacetate, β-hydroxybutyrate and acetone. These ketone bodies, except for acetone, are then used as an alternative energy source (1). The shift in energy metabolism toward fatty acid oxidation and ketone body production in KD is accompanied by a severely restricted food selection. In particular, carbohydrate-rich food groups such as cereal products, legumes, fruit and certain vegetables are very limited, which may affect the intake of essential vitamins and minerals (1, 6). The composition of the KD is often described by the ketogenic ratio, which is the ratio of fat to the total amount of carbohydrates and protein in grams. The classic KD (cKD) consists of around 80–90% of daily energy (E%) from fat and is typically implemented at a ratio of 3:1 or 4:1. More liberal variations, such as the modified Atkins diet (MAD), allow for a higher intake of protein and carbohydrates with ratios of approximately 1:1 to 2:1 and are therefore more suitable for everyday use (1, 4). Although different ratios show some comparable therapeutic effects, they differ in terms of tolerability, food selection and nutrient density. For example, Zupec-Kania et al. found that more liberal variations of KD lead to substantially more nutrient reference values being reached than stricter forms (7). The target population for KDs in adults is characterized by heterogeneity (1, 4). Differences in age, health status, energy and nutrient requirements, and dietary goals, such as therapeutic purposes or weight loss, complicate the provision of a consistent nutritional assessment (1, 4, 6). In addition, comorbidities, medication use, and individual dietary habits vary substantially, which further limits assessment of potential nutrient supply risks (4). Studies on the micronutrient supply of adults following a KD are currently limited and inconsistent. Some of these studies report reduced intake or status of specific micronutrients, particularly vitamin D, calcium, iron, zinc, and various B vitamins, in both very restrictive and more liberal forms of the KD (5, 8–10). However, observational studies show that even with added supplementation, an adequate supply of micronutrients is not necessarily achieved. Deficiencies and intakes above the recommended reference value can occur for certain micronutrients, especially when dietary supplements are used independently of nutritional therapy support (5, 11). Although a growing number of modeled or empirical studies are available for children and adolescents, corresponding data on adults remains limited (12–14). Due to the limited data available on nutrient supply in adults following a KD, this analysis aimed to evaluate the micronutrient supply of KDs in adults aged 19 and older, using optimized daily meal plans. Different ketogenic ratios (3:1, 2:1, and 1:1) were compared based on age-related reference values of the German and Austrian Nutrition Societies (DGE/ÖGE) to identify potential critical nutrients and develop recommendations for a differentiated practical implementation of KDs in adults.

## 2 Materials and methods

### 2.1 Development of meal plans

To investigate nutrient supply under different ketogenic ratios, hypothetical optimized daily meal plans were developed for adults aged between 19 and over 65. According to the DGE/ÖGE reference values, the age groups were categorized into four groups (19–24, 25–50, 51–65, and >65 years) for all three ketogenic ratios: 3:1, 2:1, and 1:1. Three daily meal plans were created for each combination of age group and ratio, resulting in a total of 36 meal plans included in the evaluation.

The daily meal plans were prepared and analyzed using the nutrition software PRODI Expert (Nutri-Science GmbH, Freiburg, Germany; version 7.3) based on the German Nutrient Database (Bundeslebensmittelschlüssel, BLS, version 3.02). For each age group, daily meal plans were first created in a 3:1 ratio, which were used as the basis for deriving the 2:1 and 1:1 ratios. Switching to the lower ratios involved specifically adjusting the amounts of fat, protein, and carbohydrates. Energy intake was based on the age-related DGE/ÖGE reference values with an assumed PAL (Physical Activity Level) of 1.6. The daily energy amount was 2,800 kcal for the age group 19 to 24, 2,700 kcal for the age group 25 to 50 and 2,500 kcal for the age group 51 and above (target range: ± 5%). Fat intake was calculated to ensure that the target ratio for each meal deviated by no more than ±0.5 from the target value (3:1, 2:1, 1:1). Carbohydrate intake was limited to a maximum of 50 g per day for ratios 3:1 and 2:1, and to a maximum of 60 g per day for ratio 1:1, regardless of age, in accordance with Low Glycemic Index Therapy (LGIT) (4). Protein intake was calculated according to respective age-related DGE/ÖGE reference values in g/day (target range: ± 5%) and was used for the ratios 3:1 and 2:1. For the 1:1 ratio, the maximum protein intake was defined as 30 E% according to LGIT (15). Table 1 shows an overview of the requirements for creating optimized meal plans. Each daily meal plan included three main meals (breakfast, lunch, and dinner). Exemplary meal plans can be found in Supplementary Table 1. Water intake was determined based on age-related DGE/ÖGE reference values for total water intake.

**Table 1:** Age-specific target values used for the ketogenic meal plans.

Age group in years*	Energy in kcal/day*	Fat	Protein in g/day*	Carbohydrate in g/day
19–24	2,800	KR of 3:1, 2:1 and 1:1	KR 3:1/2:1: 57 g/day; KR 1:1: ≤ 30 E%	KR 3:1/2:1: ≤ 50 g/day; KR 1:1: ≤ 60 g/day
25–50	2,700		KR 3:1/2:1: 57 g/day; KR 1:1: ≤ 30 E%	
51–64	2,500		KR 3:1/2:1: 55 g/day; KR 1:1: ≤ 30 E%	
≥ 65	2,500		KR 3:1/2:1: 67 g/day; KR 1:1: ≤ 30 E%	

Energy intake was based on age-specific DGE/ÖGE reference values. Fat intake was adjusted to achieve the predefined ketogenic ratios (KR) of 3:1, 2:1, and 1:1. Protein intake for KR 3:1 and 2:1 was based on age-specific DGE/ÖGE reference values, whereas for KR 1:1 protein intake was limited to ≤30 E% according to Low Glycemic Index Therapy (LGIT). Carbohydrate intake was limited to ≤50 g/day for KR 3:1 and 2:1 and to ≤60 g/day for KR 1:1 according to LGIT [modified according to (4, 15, 20)]. *According to the DGE/ÖGE reference values.

### 2.2 Data evaluation and analysis

After exporting the data to Microsoft Excel (version 2,510), all daily meal plans were checked for completion and standardization. Products with incomplete BLS nutrient information or no BLS reference were replaced with suitable BLS alternatives and adjusted accordingly to ensure a consistent data. The evaluation considered fiber, and all vitamins and minerals for which corresponding DGE/ÖGE reference values were available, for each daily meal plan. Micronutrients with missing BLS data (chromium, selenium, molybdenum) were not evaluated. Fiber and mineral supply were presented as a percentage of the respective reference values (target range: ≥ 95%). A nutrient was classified potentially critical if its supply was below 95% of the respective reference value. Descriptive statistical analyses were performed to summarize percentage deviations from reference values (mean, standard deviation, and range) using Microsoft Excel.

## 3 Results

### 3.1 Compliance with target criteria

On average the energy intake for each age group, across all meal plans and therefore all ratios, was within the target range of 95–105% of the respective reference energy. The average energy intake across all ratios was 2,756 ± 83 kcal/day for the 19–24 age group, 2,689 ± 78 kcal/day for the 25–50 age group, 2,510 ± 84 kcal/day for the 51–64 age group, and 2,464 ± 105 kcal/day for the ≥65 age group.

The average daily carbohydrate intake was 31.4 ± 2.7 g for the 3:1 ratio, 43.0 ± 5.4 g for the 2:1 ratio, and 59.1 ± 1.2 g for the 1:1 ratio. Protein intake was 0.85 ± 0.07 g/kg body weight for the 3:1 ratio, 0.88 ± 0.08 g/kg for the 2:1 ratio, and 1.55 ± 0.02 g/kg for the 1:1 ratio. Fat intake contributed 87.4 ± 0.6% to total energy for the 3:1 ratio, 84.1 ± 0.9% for the 2:1 ratio and 75.0 ± 1.5% for the 1:1 ratio. The ketogenic ratios averaged 2.87 ± 0.15 (3:1), 2.28 ± 0.16 (2:1), and 1.32 ± 0.1 (1:1), all of which were within the predefined target range. Table 2 shows the average daily intake of energy, fat, protein and carbohydrates categorized by ketogenic ratio and age group.

**Table 2:** Average daily intake of energy, fat, protein and carbohydrates for the ketogenic meal plans (*n* = 3 for each ketogenic ratio and age group) with ketogenic ratios of 3:1, 2:1 and 1:1, across the age groups according to the DGE/ÖGE reference values (19–24, 25–50, 51–64 and ≥65 years).

Energy/nutrient	Ketogenic ratio	Ages 19–24	Ages 25–50	Ages 51–64	Ages ≥ 65
Energy (mean in kcal/day ± SD)	3:1	2,740 ± 26	2,740 ± 21	2,544 ± 78	2,600 ± 22
	2:1	2,678 ± 17	2,587 ± 14	2,412 ± 5	2,415 ± 38
	1:1	2,850 ± 61	2,741 ± 5	2,574 ± 12	2,377 ± 2
Fat (mean in g/day ± SD)	3:1	267 ± 2	267 ± 2	247 ± 9	250 ± 3
	2:1	253 ± 2	243 ± 1	226 ± 1	223 ± 3
	1:1	243 ± 5	231 ± 1	213 ± 1	193 ± 1
Protein (mean in g/day ± SD)	3:1	57 ± 2	57 ± 2	55 ± 2	64 ± 1
	2:1	59 ± 0	59 ± 1	57 ± 0	67 ± 2
	1:1	109 ± 2	109 ± 2	106 ± 1	103 ± 1
Carbohydrates (mean in g/day ± SD)	3:1	33 ± 3	33 ± 3	30 ± 3	30 ± 1
	2:1	46 ± 3	45 ± 4	41 ± 4	40 ± 9
	1:1	59 ± 2	60 ± 0	60 ± 0	58 ± 2

Mean ± standard deviation (SD).

### 3.2 Nutrient supply compared to reference values

**Figure 1:**
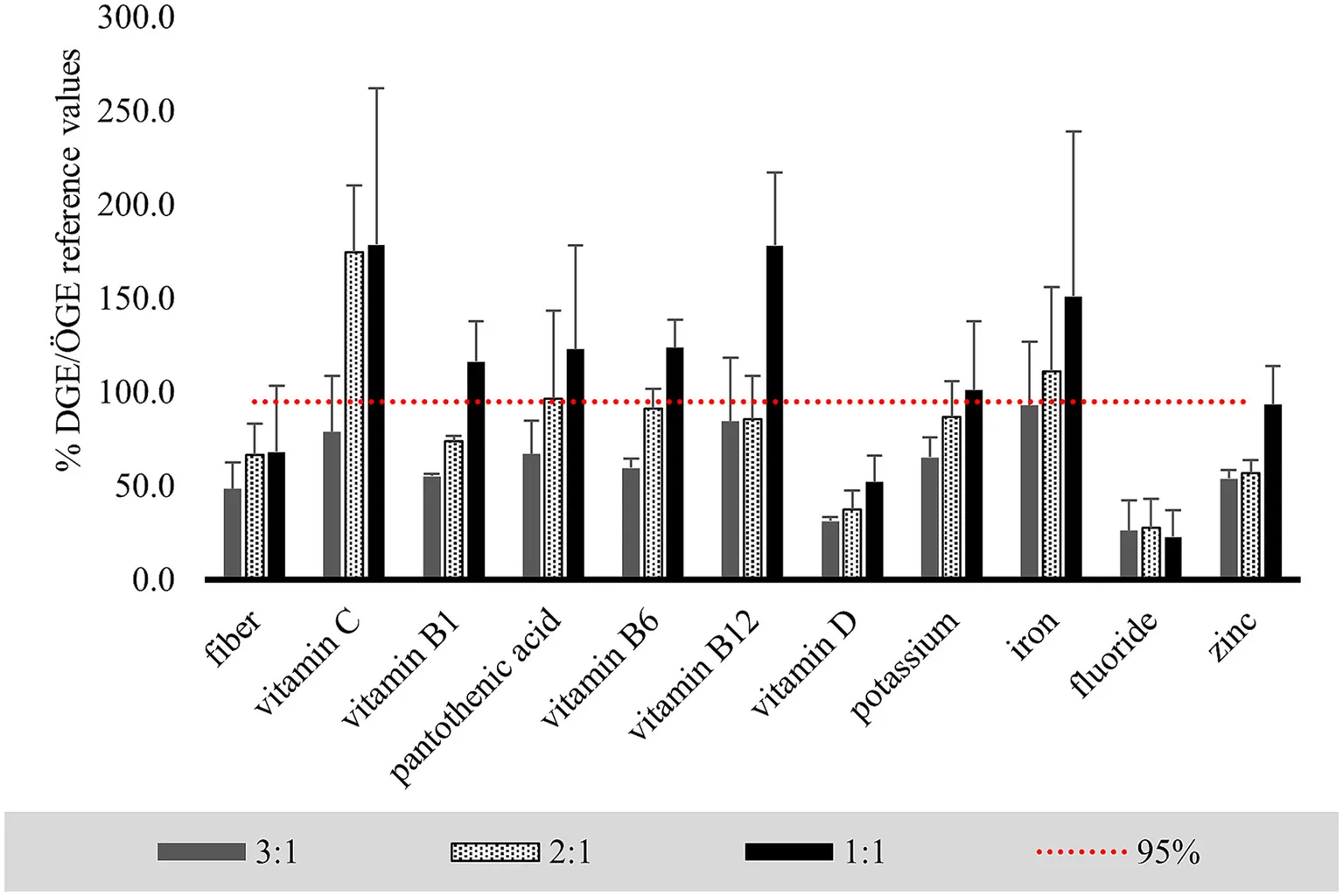
Percentage fulfillment of the DGE/ÖGE reference values for fiber and analyzed micronutrients in the 19 to 24 age group, which achieved an average of < 95% of the reference values, differentiated according to the ketogenic ratios 3:1, 2:1, and 1:1.

Among the 19–24 age group, four nutrients were below 95% of the DGE/ÖGE reference values in all three ratios, four nutrients were below 95% in two ratios (3:1 and 2:1), and three nutrients were below 95% in one ratio (3:1) (Figure 1). At all three ratios, this applied to fiber (48–68%), vitamin D (31–52%), fluoride (23–28%), and zinc (54–93%). Furthermore, at ratios of 3:1 and 2:1, vitamins B_1_ (55–74%), B_6_ (60–91%), B_12_ (85%) and potassium (65–87%) were identified as potential critical micronutrients. In the 3:1 ratio only, vitamin C (79%), pantothenic acid (67%), and iron (93%) were below 95% of the reference values.

**Figure 2:**
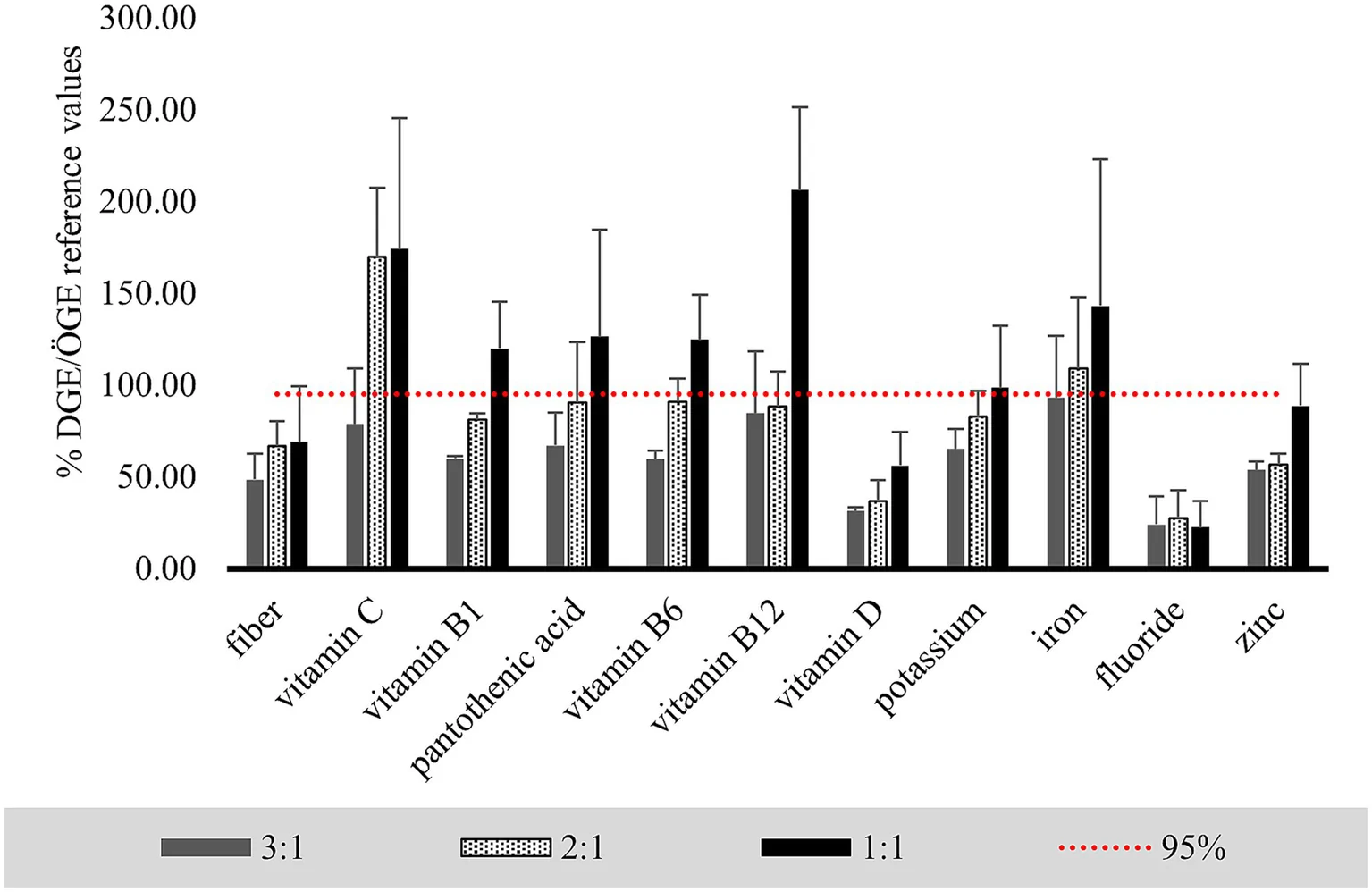
Percentage fulfillment of the DGE/ÖGE reference values for fiber and analyzed micronutrients in the 25 to 50 age group, which achieved an average of < 95% of the reference values, differentiated according to the ketogenic ratios 3:1, 2:1, and 1:1.

Among the 25–50 age group, four nutrients were below 95% of the DGE/ÖGE reference values in all three ratios, five nutrients were below 95% in two ratios (3:1 and 2:1), and two nutrients were below 95% in one ratio (3:1) (Figure 2). In all three ratios, this concerned fiber (48–69%), vitamin D (31–56%), fluoride (22–27%), and zinc (54–89%). In the two ratios 3:1 and 2:1, this additional applied to vitamin B_1_ (60–81%), pantothenic acid (67–90%), vitamin B_6_ (60–91%), vitamin B_12_ (85–88%), and potassium (65–83%). Only in the 3:1 ratio, were vitamin C (79%) and iron (93%) below 95% of the reference values.

**Figure 3:**
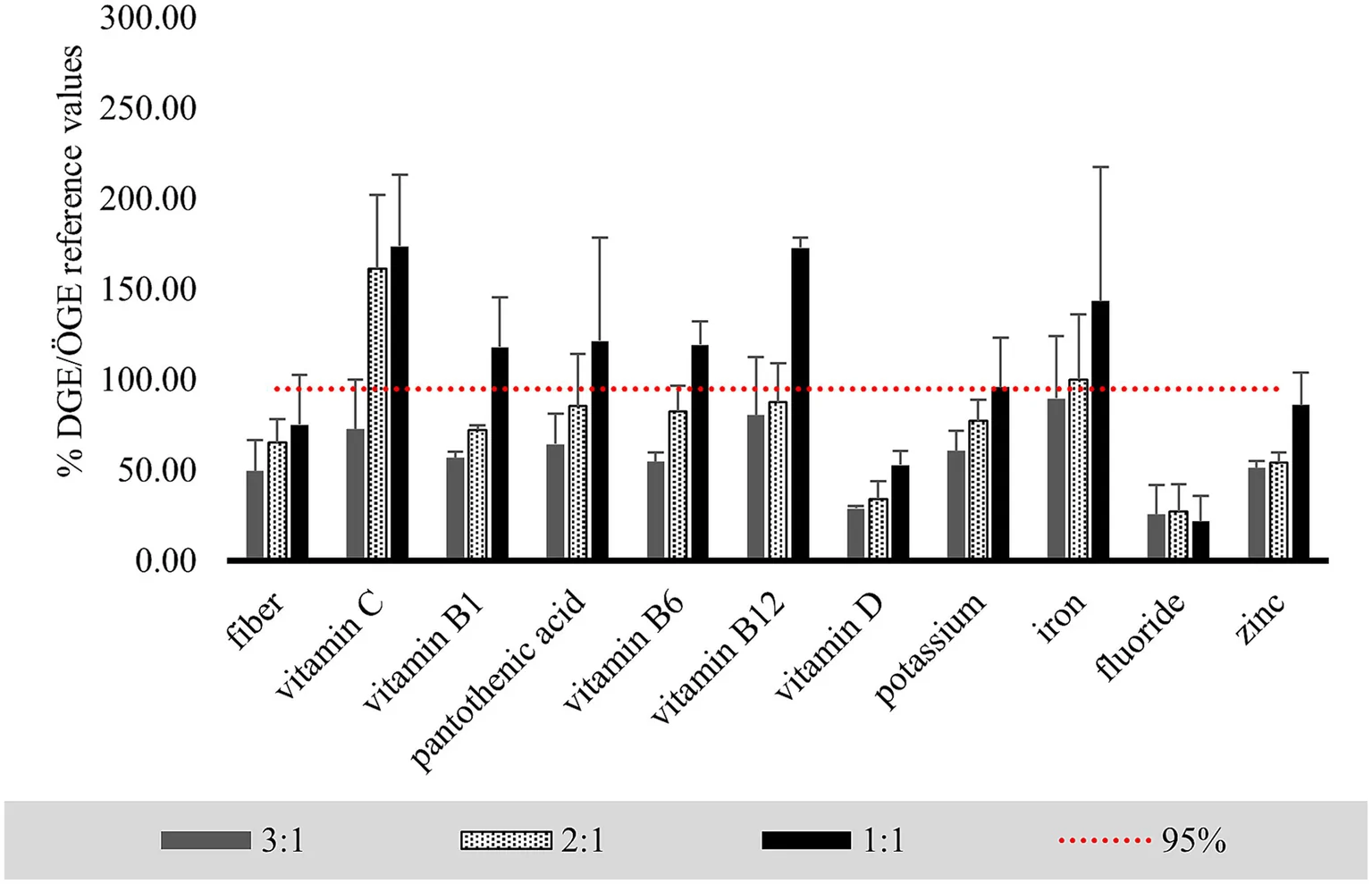
Percentage fulfillment of the DGE/ÖGE reference values for fiber and analyzed micronutrients in the 51 to 64 age group, which achieved an average of < 95% of the reference values, differentiated according to the ketogenic ratios 3:1, 2:1, and 1:1.

For the 51–64 age group, four nutrients were below 95% of the DGE/ÖGE reference values in all three ratios, five nutrients were below 95% in two ratios (3:1 and 2:1), and two nutrients were below 95% in one ratio (3:1) (Figure 3). Fiber (49–75%), vitamin D (29–53%), fluoride (22–27%), and zinc (51–86%) were below 95% of the reference values in all three ratios. In the 3:1 and 2:1 ratio vitamin B_1_ (57–72%), pantothenic acid (64–85%), vitamin B_6_ (55–83%), vitamin B_12_ (81–87%), and potassium (61–77%) were also below 95% of the reference values. In the 3:1 ratio only, the reference value for vitamin C (73%) and iron (90%) was not reached.

**Figure 4:**
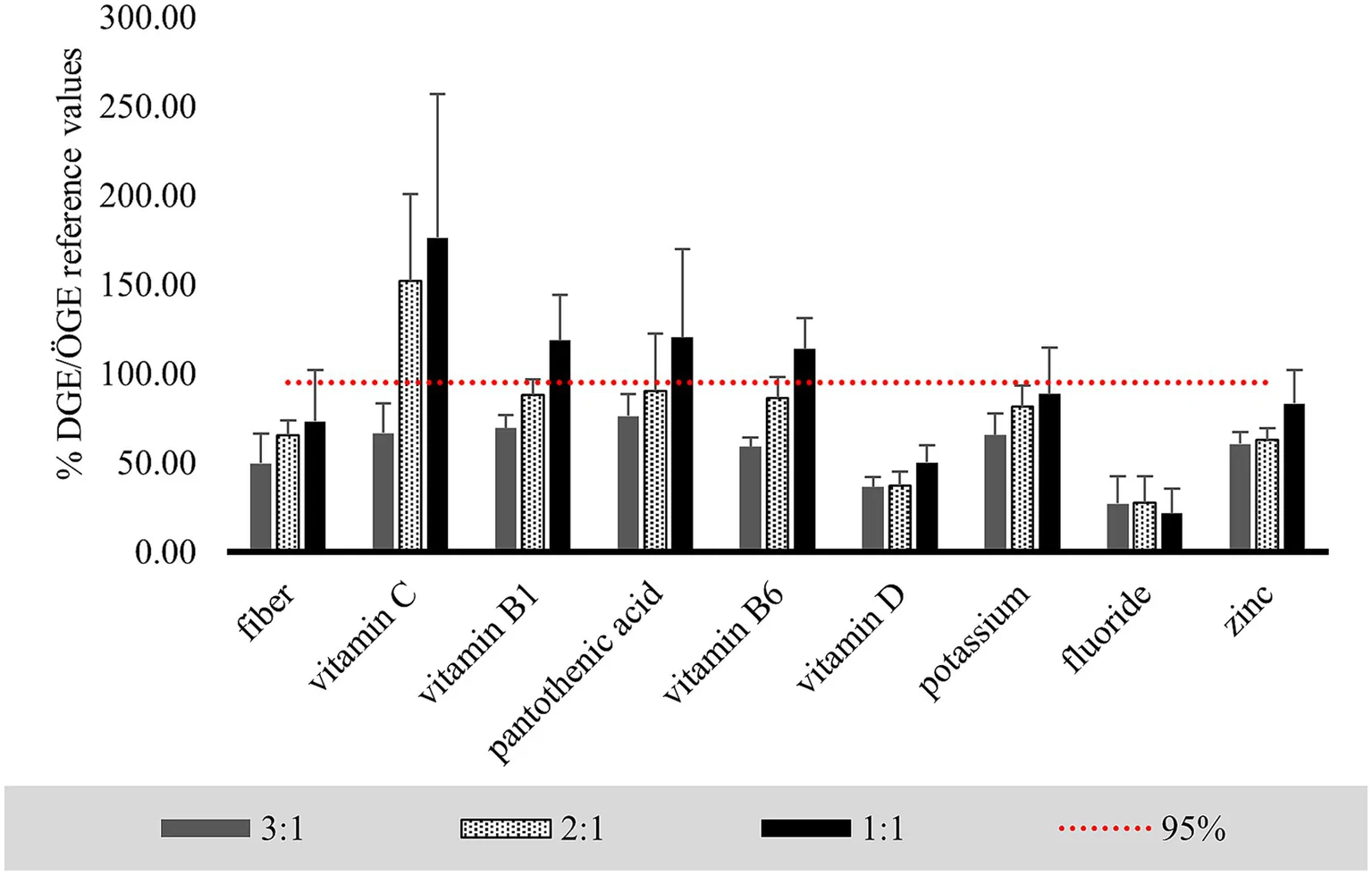
Percentage fulfillment of the DGE/ÖGE reference values for fiber and analyzed micronutrients in the age group 65+, which achieved an average of < 95% of the reference values, differentiated according to the ketogenic ratios 3:1, 2:1, and 1:1.

In the 65 + age group, five nutrients were below 95% of the DGE/ÖGE reference values in all three ratios, three nutrients were below 95% in two ratios (3:1 and 2:1), and one nutrient was below 95% in one ratio (3:1) (Figure 4). In all three ratios, this applied to fiber (50–73%), vitamin D (36–50%), potassium (66–89%), fluoride (22–28%), and zinc (61–83%). In the 3:1 and 2:1 ratios vitamin B_1_ (70–88%), pantothenic acid (76–90%), and vitamin B_6_ (59–86%) were identified as potentially critical vitamins. In the 3:1 ratio only, vitamin C (67%) was below 95% of the reference value. Supplementary Tables 2–4 provide an overview of the calculated daily nutrient intake for ratios of 3:1, 2:1 and 1:1 across all age groups, as well as the mean percentage achievement of DGE/ÖGE reference values.

### 3.3 Number of daily meal plans below the reference values

Regardless of age group, all daily meal plans in the 3:1 ratio (*n* = 12) were below the reference value of 95% for eight nutrients. These nutrients included fiber, vitamin B_1_, pantothenic acid, vitamin B_6_, vitamin D, potassium, fluoride, and zinc. Three other nutrients remained below 95% in at least half of the daily meal plans, including vitamin C (9/12 daily meal plans; 75%), vitamin B_12_ (8/12 daily meal plans; 67%), and iron (8/12 daily meal plans; 67%). In contrast, four nutrients were below the 95% reference value in all daily plans in the 2:1 ratio (fiber, vitamin D, fluoride, and zinc), and five other nutrients were below the threshold in at least half of the daily meal plans. These were vitamin B_1_ (11/12 daily meal plans; 92%), pantothenic acid (8/12 daily meal plans; 67%), vitamin B_6_ (7/12 daily meal plans; 58%), and potassium (11/12 daily meal plans; 92%). In the 1:1 ratio, all daily meal plans were below the 95% threshold for the two micronutrients vitamin D and fluoride. Two other nutrients were below 95% in at least half of the daily plans, specifically fiber (8/12 daily meal plans; 67%) and zinc (8/12 daily meal plans; 67%) (see Table 3).

**Table 3:** Number and percentage of daily plans with nutrients below 95% of the DGE/ÖGE reference values, subdivided by ketogenic ratio (3:1, 2:1, 1:1; each *n* = 12).

Nutrient	3:1 (*n*, %)	2:1 (*n*, %)	1:1 (*n*, %)
Fiber	12 (100)	12 (100)	8 (67)
Vitamin C	9 (75)	0 (0)	0 (0)
Vitamin B₁	12 (100)	11 (92)	2 (17)
Pantothenic acid	12 (100)	8 (67)	5 (42)
Vitamin B₆	12 (100)	7 (58)	1 (8)
Biotin	3 (25)	2 (17)	0 (0)
Folate	4 (33)	0 (0)	0 (0)
Vitamin B₁₂	8 (67)	8 (67)	0 (0)
Vitamin D	12 (100)	12 (100)	12 (100)
Sodium	0 (0)	2 (17)	0 (0)
Chloride	3 (25)	4 (33)	0 (0)
Potassium	12 (100)	11 (92)	5 (42)
Iron	8 (67)	5 (42)	4 (33)
Fluoride	12 (100)	12 (100)	12 (100)
Zinc	12 (100)	12 (100)	8 (67)

## 4 Discussion

The results suggest a ratio-dependent pattern of micronutrient supply in adults. The 3:1 ratio, the most restrictive, showed distinct potential nutrient deficits across all age groups. In contrast, the 2:1 ratio exhibited moderate improvement, but still contained relevant nutrient gaps. Although the most liberal 1:1 ratio largely achieved sufficient values for most nutrients, certain nutrients remain potential critical regardless of the ratio. These findings are consistent with those of studies based on dietary protocols, modeled meal plans, or observational studies involving children, adolescents, and adults. Previous studies have showed that stricter ketogenic ratios are associated with lower micronutrient density due to severely restricted food selection, whereas more liberal ketogenic approaches allow for a wider selection of food and thus improved nutrient intake (4, 8–10). In the present daily meal plans, fiber intake was below the age-related reference values for all examined ketogenic ratios, with the lowest supply achieved at a ratio of 3:1. An improvement was observed with increased liberalization of the diet, but fiber intake remained insufficient on average, even at a 1:1 ratio. Similar results have been reported in studies investigating low-carbohydrate diets and KDs in adults. Reduced fiber intake was primarily attributed to the exclusion of fiber-rich foods such as whole grains, legumes, and various types of fruit (8, 10, 16). Conversely, analyses of less restrictive ketogenic approaches indicate that higher fiber intake can be achieved by incorporating fiber-rich, low-carbohydrate foods, such as non-starchy vegetables, nuts, seeds, and functional fiber products, into the diet. However, this requires intentional food-based planning (4, 12).

For B vitamins, the daily meal plans demonstrated a ratio-dependent supply pattern. KDs were particularly likely to have insufficient values of vitamin B_1_, pantothenic acid, vitamin B_6_, and vitamin B_12_, whereas a less restrictive form of KD showed a gradual progression toward the recommended reference values. These results are in line with observations in adults on low-carbohydrate diets and KDs. For example, a cross-sectional analysis of adults with erythrocyte-based determination of functional thiamine status revealed reduced vitamin B_1_ status in low-carbohydrate diets and KDs (5). Furthermore, a model-based analysis of ketogenic daily meal plans for adults showed that several B vitamins, including pantothenic acid, may be supplied in smaller quantities when food choices are limited (8). Primarily, this is attributed to reduced consumption of vitamin-rich plant-based foods and certain animal protein sources, such as dairy products and lean meat, which can only be consumed in limited quantities under strict ketogenic conditions (10). Calculated intake levels of vitamin C were below the 95% threshold in more restrictive diets, but sufficient values were achieved in less restrictive ketogenic ratios. Restrictive meal plans, like 3:1, imposed the greatest restrictions on carbohydrate intake, accompanied by a lower proportion of fruit and vegetables, and therefore vitamin C. Evaluations of low-carbohydrate diets and KDs in adults illustrate that the supply of vitamin C depends strongly on food selection and the option for food variety (4, 10).

Existing literature repeatedly described vitamin D as a potentially critical micronutrient in relation to ketogenic and severely carbohydrate-restricted diets. Clinical studies and international consensus papers suggest that adult patients on KDs have low intake levels and suboptimal vitamin D serum levels (4, 17). These findings align with the results of the proposed optimized daily meal plans, in which vitamin D was consistently below the reference values. However, insufficient vitamin D intake is not specific to KDs. National consumption data shows that even with a normal mixed diet, adults in Germany already consume significantly less than the recommended daily intake of vitamin D on a median basis. This is mainly due to the very limited availability of foods rich in vitamin D (18). At the European level, vitamin D is also considered as critical, regardless of dietary patterns, since endogenous synthesis through sun exposure is the primary source of supply and sufficient intake through food alone is usually not achieved (19).

In addition to vitamins, there are also indications of a potentially insufficient supplies of certain minerals in KDs. Levran et al. based their findings on dietary records in adults on low-carbohydrate diets and reported low intakes of potassium, iron, and zinc, primarily associated with reduced consumption of plant-based foods (10). Christodoulides et al. conducted a model-based nutritional analysis of KDs in adults and demonstrated that several minerals, including potassium and zinc, consistently fall below reference values when food choices are limited (8). These results are consistent with the present analysis, in which potassium and zinc were identified as potentially critical, particularly in more restrictive ratios (3:1 and 2:1), while iron fell below the reference values only in the strictest 3:1 ratio. Fluoride supply was considerably below the reference values in all age groups and ratios. No comparable studies on fluoride intake in adults on KDs are yet available. Overall, the results emphasize that the minerals potassium, zinc, and fluoride require special consideration for adults on a KD.

This analysis was based on age-related reference values and used male adults as an example, due to their higher energy intake according to the reference values. However, gender-specific differences must be considered when interpreting the results. For women, the age-dependent reference values for energy requirements are approximately 20–24% lower than those for men across all considered age groups (20). At the same time, the reference values for several micronutrients identified as potentially critical in this analysis, including pantothenic acid, vitamin B_12_, and potassium, are defined independently of gender. This means that women must achieve these reference values with a lower energy intake, which could potentially further increase the risk of inadequate nutrient intake under restrictive KDs. This effect is particularly pronounced for iron, for which the reference value age is up to 50% higher for women of reproductive age than for men (21). Against this background, the observed supply gaps for individual micronutrients in adult women are expected to increase, especially with stricter ketogenic ratios. These factors must be considered in nutritional therapy practice, with individual assessments and recommendations provided.

Although the general patterns of potential micronutrient deficits were similar across age groups, older age is associated with physiological changes that can affect the absorption and supply of nutrients. It is well-known that the absorption of vitamin B_12_ decreases with age, partly due to reduced secretion of gastric acid and intrinsic factor, which puts older adults at greater risk of subclinical deficiencies (22, 23). Additionally, the skin’s capacity to synthesize vitamin D and exposure to sunlight both decrease with age, resulting in lower vitamin D supply (24, 25). Against this background, the findings for older adults are particularly relevant in nutrition therapy.

This analysis has some limitations, which should be considered when interpreting the results. Firstly, the analysis is based on optimized, hypothetical daily meal plans. Therefore, it does not reflect the heterogeneity of eating behaviors of the adult population or individual goals of KDs, which can vary considerably from person to person (2). Secondly, aspects such as different indications, comorbidities, medication intake and resulting dietary adjustments could not be taken into account. The rather minor differences between the age groups must be interpreted in the context of similar energy requirements among adults, resulting in largely comparable daily schedules. Finally, the potentially critical nutrients identified depend largely on food choices. Thus, while the results reflect the structural characteristics of KDs, they do not allow for an evaluation of individual dietary strategies, which depend largely on compliance and the therapeutic goal (6, 26). Furthermore, the BLS does not provide any information on carnitine (27). This may be relevant in the context of a KD, but it is not automatically associated with clinical symptoms (28). However, the evaluated hypothetical meal plans highlight the known risks of potential deficiencies in water-soluble vitamins and certain minerals associated with KDs, particularly under strict ketogenic conditions.

## 5 Conclusion

The analysis of hypothetical meal plans showed that the supply of micronutrient in KDs for adults depends considerably on the chosen ketogenic ratio. While the 3:1 ratio resulted in pronounced deficits of several vitamins and minerals across all age groups, the 2:1 ratio showed moderate improvement and the 1:1 ratio demonstrated strong tendency to achieve reference values. The reason for this is the limited food selection in restrictive KDs. Although differences between age groups were small, physiological changes in older people must be also considered. In addition to careful food selection, nutritional therapy is necessary in order to identify and treat nutrient deficiencies early on and on an individual basis.

## Data Availability

The raw data supporting the conclusions of this article will be made available by the authors, without undue reservation.
