# Supplementary material for: Analysis of critical nutrients in ketogenic diets for adults based on optimized hypothetical meal plans
Source: Front Nutr. 2026 Sep 11;13:1829975. doi: 10.3389/fnut.2026.1829975 (PMC13614011; doi:10.3389/fnut.2026.1829975)
Supplement: Supplementary file 1 [file Data_Sheet_1.PDF]

Supplementary Material

1 Supplementary Tables

Supplementary Table 1. Examples of optimized, hypothetical ketogenic meal plans based on a 2:1 ketogenic ratio. Categorized by age groups (19-24, 25–50, 51–64 and ≥65 years) and by meal type (breakfast, lunch and dinner).

meal	ages 19 – 24	ages 25 – 50	ages 51 – 64	ages ≥ 65
**breakfast**	*Pancake*<br><br>mascarpone, almond flour, baking powder, egg, canola oil, almond butter, strawberries, coffee, sparkling water	*Muesli*<br><br>brazil nuts, macadamia nuts, sunflower seeds, yogurt, soy milk, flaxseed oil, blueberries, banana, coffee, sparkling water	*Raspberry milkshake*<br><br>raspberries, lemon juice, coconut milk, cream, canola oil, coffee, sparkling water	*Pancake*<br><br>mascarpone, almond flour, baking powder, egg, canola oil, almond butter, strawberries, coffee, sparkling water
**lunch**	*Vegetable tagliatelle*<br><br>zucchini, cucumber, walnuts, hazelnut oil, canola oil, crème fraîche, goat cheese, lemon juice, salmon, sparkling water	*Tomato quiche*<br><br>almond flour, Parmesan, flaxseeds, canola oil, eggs, butter, mascarpone, onions, tomatoes, bell peppers, arugula, sparkling water	*Spinach casserole*<br><br>olive oil, spinach, basil, mascarpone, crème fraîche, soy cream, Parmesan, egg, canned tomatoes in oil, mushrooms, sparkling water	*Vegetable tagliatelle*<br><br>zucchini, cucumber, walnuts, hazelnut oil, canola oil, crème fraîche, goat cheese, lemon juice, salmon, sparkling water
**dinner**	*Avocado boats*<br><br>tomatoes, avocado, feta, corn, lemon juice, gouda,	*Tuna wraps*<br><br>eggs, mascarpone, iodized table salt with added fluoride and folic acid,	*Bread with tomato salsa*<br><br>almond flour, flaxseed, baking powder, iodized table salt with added	*Avocado boats*<br><br>tomatoes, avocado, feta, corn, lemon juice, gouda,

	canola oil, mascarpone, sparkling water	baking powder, mayonnaise, yogurt, canned tuna in oil, lamb's lettuce, cucumber, parsley, pumpkin seeds, pumpkin seed oil, sparkling water	fluoride and folic acid, wine vinegar, egg, butter, tomatoes, onions, scallions, garlic, salami, olive oil, sparkling water	canola oil, mascarpone, sparkling water

Supplementary Table 2. Mean and standard deviation (SD) of the calculated daily nutrients for the optimized hypothetical ketogenic meal plans with a ratio of 3:1 across four age groups (19-24, 25–50, 51–64 and ≥65 years). Values in brackets represent the mean percentage ± SD of achievement of the DGE/ÖGE reference values.

**nutrient**	**ages 19 – 24**	**ages 25 – 50**	**ages 51 – 64**	**ages ≥ 65**
**fiber (g)**	19 ± 6 (48 ± 14)	19 ± 5 (48 ± 14)	18 ± 6 (49 ± 17)	19 ± 6 (50 ± 17)
**vitamin C (mg)**	87 ± 33 (79 ± 30)	87 ± 27 (79 ± 30)	80 ± 30 (73 ± 27)	73 ± 19 (67 ± 17)
**vitamin B_1 (mg)**	1 ± 0 (55 ± 1)	1 ± 0 (60 ± 2)	1 ± 0 (57 ± 4)	1 ± 0 (70 ± 7)
**vitamin B_2 (mg)**	1 ± 0 (105 ± 4)	1 ± 0 (105 ± 4)	1 ± 0 (106 ± 4)	2 ± 0 (119 ± 11)
**vitamin B_3 (mg)**	19 ± 1 (113 ± 6)	19 ± 1 (128 ± 7)	18 ± 1 (122 ± 6)	21 ± 1 (149 ± 8)
**pantothenic acid (mg)**	3 ± 1 (67 ± 18)	3 ± 1 (67 ± 18)	3 ± 1 (64 ± 17)	4 ± 1 (76 ± 12)
**vitamin B_6 (mg)**	1 ± 0 (60 ± 5)	1 ± 0 (60 ± 5)	1 ± 0 (55 ± 5)	1 ± 0 (59 ± 5)
**biotin (µg)**	49 ± 14 (121 ± 36)	49 ± 12 (121 ± 36)	47 ± 14 (118 ± 36)	57 ± 15 (143 ± 36)
**folate (µg)**	440 ± 184 (147 ± 61)	423 ± 139 (141 ± 57)	421 ± 181 (140 ± 60)	449 ± 171 (150 ± 57)
**vitamin B_{12} (µg)**	3 ± 1 (85 ± 34)	3 ± 1 (85 ± 34)	3 ± 1 (81 ± 32)	4 ± 1 (103 ± 34)
**retinol activity equivalent (µg)**	1788 ± 287 (210 ± 34)	1788 ± 234 (210 ± 34)	1670 ± 227 (196 ± 27)	1756 ± 172 (220 ± 21)
**vitamin D (µg)**	6 ± 0 (31 ± 2)	6 ± 0 (31 ± 2)	6 ± 0 (29 ± 1)	7 ± 1 (36 ± 6)
**vitamin E (mg)**	37 ± 4 (464 ± 45)	37 ± 3 (464 ± 45)	35 ± 3 (435 ± 40)	35 ± 1 (442 ± 17)
**vitamin K (µg)**	273 ± 211 (391 ± 302)	273 ± 172 (391 ± 302)	254 ± 194 (317 ± 242)	257 ± 202 (322 ± 252)
**sodium (mg)**	1886 ± 270 (126 ± 18)	1810 ± 225 (121 ± 18)	1754 ± 248 (117 ± 17)	1964 ± 198 (131 ± 13)
**chloride (mg)**	2425 ± 406 (105 ± 18)	2321 ± 291 (101 ± 16)	2255 ± 355 (98 ± 15)	2511 ± 239 (109 ± 10)
**potassium (mg)**	2608 ± 429 (65 ± 11)	2607 ± 350 (65 ± 11)	2432 ± 442 (61 ± 11)	2629 ± 489 (66 ± 12)
**calcium (mg)**	1527 ± 189 (153 ± 19)	1491 ± 155 (149 ± 19)	1321 ± 196 (132 ± 20)	1449 ± 369 (145 ± 37)
**phosphorus (mg)**	1336 ± 27 (243 ± 5)	1336 ± 22 (243 ± 5)	1273 ± 24 (232 ± 4)	1453 ± 115 (264 ± 21)
**magnesium (mg)**	561 ± 88 (160 ± 25)	550 ± 72 (157 ± 25)	502 ± 91 (143 ± 26)	528 ± 122 (151 ± 35)
**iron (mg)**	10 ± 4 (93 ± 34)	10 ± 3 (93 ± 34)	10 ± 4 (90 ± 34)	11 ± 4 (100 ± 40)
**iodine (µg)**	265 ± 31 (177 ± 21)	261 ± 32 (174 ± 26)	248 ± 40 (166 ± 26)	242 ± 37 (161 ± 24)
**fluoride (mg)**	1 ± 1 (26 ± 16)	1 ± 0 (24 ± 15)	1 ± 1 (26 ± 16)	1 ± 1 (27 ± 15)
**zinc (mg)**	8 ± 1 (54 ± 5)	8 ± 1 (54 ± 5)	7 ± 1 (51 ± 4)	8 ± 1 (61 ± 6)
**copper (mg)[a]**	2 ± 0	2 ± 0	2 ± 0	2 ± 1
**manganese (mg)[b]**	4 ± 2	4 ± 2	4 ± 2	4 ± 2

[a] The recommended daily intake of copper is between 1 and 1.5 mg/day. Therefore, exact percentages cannot be determined.
[b] The recommended daily intake of manganese is between 2 and 5 mg/day. Therefore, exact percentages cannot be determined.

Supplementary Table 3. Mean and standard deviation (SD) of the calculated daily nutrients for the optimized hypothetical ketogenic meal plans with a ratio of 2:1 across four age groups (19-24, 25–50, 51–64 and ≥65 years). Values in brackets represent the mean percentage ± SD of achievement of the DGE/ÖGE reference values.

nutrient	ages 19 – 24	ages 25 – 50	ages 51 – 64	ages ≥ 65
**fiber (g)**	26 ± 6 (67 ± 17)	25 ± 5 (67 ± 13)	23 ± 5 (65 ± 13)	23 ± 2 (66 ± 8)
**vitamin C (mg)**	192 ± 39 (174 ± 36)	187 ± 41 (170 ± 37)	178 ± 45 (161 ± 41)	167 ± 54 (152 ± 49)
**vitamin B_1 (mg)**	1 ± 0 (74 ± 3)	1 ± 0 (81 ± 4)	1 ± 0 (72 ± 3)	1 ± 0 (88 ± 9)
**vitamin B_2 (mg)**	2 ± 0 (127 ± 19)	2 ± 0 (124 ± 13)	2 ± 0 (122 ± 6)	2 ± 0 (132 ± 9)
**vitamin B_3 (mg)**	24 ± 3 (141 ± 18)	23 ± 2 (154 ± 11)	22 ± 1 (145 ± 5)	25 ± 1 (177 ± 10)
**pantothenic acid (mg)**	5 ± 2 (97 ± 47)	5 ± 2 (90 ± 33)	4 ± 1 (85 ± 29)	5 ± 2 (90 ± 32)
**vitamin B_6 (mg)**	1 ± 0 (91 ± 11)	1 ± 0 (91 ± 13)	1 ± 0 (83 ± 14)	1 ± 0 (86 ± 12)
**biotin (µg)**	62 ± 25 (154 ± 62)	61 ± 19 (151 ± 47)	55 ± 18 (138 ± 45)	61 ± 25 (153 ± 63)
**folate (µg)**	540 ± 182 (180 ± 61)	534 ± 168 (178 ± 56)	504 ± 162 (168 ± 54)	521 ± 166 (174 ± 55)
**vitamin B_{12} (µg)**	3 ± 1 (85 ± 23)	4 ± 1 (88 ± 19)	3 ± 1 (87 ± 22)	4 ± 1 (99 ± 22)
**retinol activity equivalent (µg)**	1734 ± 197 (204 ± 23)	1750 ± 252 (206 ± 30)	1609 ± 161 (189 ± 19)	1674 ± 130 (209 ± 16)
**vitamin D (µg)**	8 ± 2 (38 ± 10)	7 ± 2 (37 ± 11)	7 ± 2 (34 ± 10)	7 ± 2 (37 ± 8)
**vitamin E (mg)**	38 ± 1 (481 ± 10)	37 ± 1 (461 ± 13)	33 ± 1 (418 ± 17)	34 ± 2 (424 ± 29)
**vitamin K (µg)**	355 ± 266 (508 ± 381)	339 ± 251 (484 ± 358)	307 ± 221 (383 ± 277)	328 ± 275 (410 ± 344)
**sodium (mg)**	1960 ± 549 (131 ± 37)	1826 ± 425 (122 ± 28)	1765 ± 416 (118 ± 28)	2015 ± 313 (134 ± 21)
**chloride (mg)**	2477 ± 689 (108 ± 30)	2406 ± 679 (105 ± 30)	2359 ± 695 (103 ± 30)	2668 ± 572 (116 ± 25)
**potassium (mg)**	3477 ± 749 (87 ± 19)	3312 ± 559 (83 ± 14)	3082 ± 473 (77 ± 12)	3259 ± 480 (81 ± 12)
**calcium (mg)**	1396 ± 53 (140 ± 5)	1350 ± 20 (135 ± 2)	1254 ± 83 (125 ± 8)	1380 ± 238 (138 ± 24)
**phosphorus (mg)**	1473 ± 199 (268 ± 36)	1369 ± 22 (249 ± 4)	1317 ± 14 (239 ± 3)	1479 ± 86 (269 ± 16)
**magnesium (mg)**	565 ± 88 (161 ± 25)	551 ± 78 (157 ± 22)	486 ± 71 (139 ± 20)	528 ± 77 (151 ± 22)
**iron (mg)**	12 ± 5 (111 ± 45)	12 ± 4 (109 ± 39)	11 ± 4 (100 ± 36)	12 ± 5 (111 ± 43)
**iodine (µg)**	252 ± 36 (168 ± 24)	248 ± 40 (165 ± 27)	232 ± 38 (155 ± 25)	240 ± 44 (160 ± 29)
**fluoride (mg)**	1 ± 1 (27 ± 15)	1 ± 1 (28 ± 15)	1 ± 1 (27 ± 15)	1 ± 1 (28 ± 15)
**zinc (mg)**	8 ± 1 (57 ± 7)	8 ± 1 (57 ± 6)	8 ± 1 (54 ± 6)	9 ± 1 (63 ± 7)
**copper (mg)[a]**	2 ± 1	2 ± 0	2 ± 0	2 ± 0
**manganese (mg)[b]**	5 ± 2	5 ± 2	4 ± 2	5 ± 2

[a] The recommended daily intake of copper is between 1 and 1.5 mg/day. Therefore, exact percentages cannot be determined.
[b] The recommended daily intake of manganese is between 2 and 5 mg/day. Therefore, exact percentages cannot be determined.

Supplementary Table 4. Mean and standard deviation (SD) of the calculated daily nutrients for the optimized hypothetical ketogenic meal plans with a ratio of 1:1 across four age groups (19-24, 25–50, 51–64 and ≥65 years). Values in brackets represent the mean percentage ± SD of achievement of the DGE/ÖGE reference values.

nutrient	ages 19 – 24	ages 25 – 50	ages 51 – 64	ages ≥ 65
**fiber (g)**	28 ± 15 (68 ± 35)	28 ± 12 (69 ± 31)	28 ± 10 (75 ± 28)	25 ± 10 (73 ± 29)
**vitamin C (mg)**	196 ± 92 (179 ± 84)	191 ± 79 (174 ± 71)	191 ± 44 (174 ± 40)	194 ± 89 (176 ± 81)
**vitamin B_1 (mg)**	2 ± 0 (116 ± 22)	1 ± 0 (120 ± 26)	1 ± 0 (118 ± 27)	1 ± 0 (119 ± 25)
**vitamin B_2 (mg)**	2 ± 0 (158 ± 17)	2 ± 0 (160 ± 24)	2 ± 0 (165 ± 33)	2 ± 0 (168 ± 24)
**vitamin B_3 (mg)**	44 ± 2 (256 ± 10)	45 ± 3 (298 ± 20)	45 ± 4 (302 ± 29)	41 ± 2 (294 ± 13)
**pantothenic acid (mg)**	6 ± 3 (123 ± 56)	6 ± 3 (126 ± 58)	6 ± 3 (121 ± 57)	6 ± 2 (121 ± 50)
**vitamin B_6 (mg)**	2 ± 0 (124 ± 15)	2 ± 0 (125 ± 24)	2 ± 0 (119 ± 13)	2 ± 0 (114 ± 17)
**biotin (µg)**	93 ± 42 (231 ± 106)	91 ± 44 (227 ± 109)	90 ± 43 (224 ± 108)	85 ± 36 (214 ± 91)
**folate (µg)**	570 ± 286 (190 ± 95)	553 ± 265 (184 ± 88)	546 ± 249 (182 ± 83)	525 ± 237 (175 ± 79)
**vitamin B_{12} (µg)**	7 ± 2 (178 ± 39)	8 ± 2 (206 ± 45)	7 ± 0 (173 ± 6)	7 ± 1 (187 ± 13)
**retinol activity equivalent (µg)**	1653 ± 178 (194 ± 21)	1568 ± 253 (184 ± 30)	1540 ± 213 (181 ± 25)	1502 ± 198 (188 ± 25)
**vitamin D (µg)**	10 ± 3 (52 ± 14)	11 ± 4 (56 ± 18)	11 ± 2 (53 ± 8)	10 ± 2 (50 ± 10)
**vitamin E (mg)**	41 ± 5 (515 ± 63)	39 ± 5 (484 ± 57)	38 ± 7 (477 ± 91)	32 ± 5 (401 ± 63)
**vitamin K (µg)**	400 ± 419 (571 ± 598)	351 ± 333 (502 ± 475)	328 ± 324 (410 ± 405)	283 ± 267 (354 ± 334)
**sodium (mg)**	2353 ± 672 (157 ± 45)	2282 ± 722 (152 ± 48)	2123 ± 656 (142 ± 44)	2238 ± 739 (149 ± 49)
**chloride (mg)**	3398 ± 782 (148 ± 34)	3331 ± 1001 (145 ± 44)	3152 ± 687 (137 ± 30)	3240 ± 829 (141 ± 36)
**potassium (mg)**	4045 ± 1472 (101 ± 37)	3942 ± 1341 (99 ± 34)	3836 ± 1085 (96 ± 27)	3545 ± 1047 (89 ± 26)
**calcium (mg)**	1864 ± 340 (186 ± 34)	1727 ± 105 (173 ± 10)	1549 ± 333 (155 ± 33)	1632 ± 281 (163 ± 28)
**phosphorus (mg)**	2207 ± 87 (401 ± 16)	2168 ± 115 (394 ± 21)	2106 ± 48 (383 ± 9)	2058 ± 115 (374 ± 21)
**magnesium (mg)**	703 ± 146 (201 ± 42)	654 ± 140 (187 ± 40)	626 ± 100 (179 ± 29)	577 ± 103 (165 ± 29)
**iron (mg)**	17 ± 10 (151 ± 88)	16 ± 9 (143 ± 80)	16 ± 8 (144 ± 74)	14 ± 7 (129 ± 64)
**iodine (µg)**	257 ± 126 (171 ± 84)	254 ± 120 (169 ± 80)	269 ± 157 (179 ± 105)	251 ± 113 (167 ± 75)
**fluoride (mg)**	1 ± 0 (23 ± 14)	1 ± 1 (22 ± 14)	1 ± 0 (22 ± 14)	1 ± 0 (22 ± 14)
**zinc (mg)**	13 ± 3 (93 ± 20)	12 ± 3 (89 ± 23)	12 ± 3 (86 ± 18)	12 ± 3 (83 ± 19)
**copper (mg)[a]**	3 ± 1	3 ± 1	3 ± 1	2 ± 1
**manganese (mg)[b]**	6 ± 2	6 ± 2	7 ± 3	6 ± 2

[a] The recommended daily intake of copper is between 1 and 1.5 mg/day. Therefore, exact percentages cannot be determined.
[b] The recommended daily intake of manganese is between 2 and 5 mg/day. Therefore, exact percentages cannot be determined.
